# Near-complete genome sequences of a rice necrosis mosaic virus isolate infecting rice in Argentina

**DOI:** 10.1128/mra.00598-25

**Published:** 2025-11-24

**Authors:** V. E. Solís, M. Bangratz, M. F. Brugo Carivali, A. Comte, C. E. Luciani, S. Lacombe, M. L. Fontana, D. Filloux, M. I. Pachecoy, E. Fernandez, P. M. Dirchwolf, J. O. A. Ayala, C. Julian, R. D. Kruger, E. Hébrard, F. D. Fernandez, M. C. Perotto, P. Roumagnac, S. A. Gutiérrez, N. Poulicard, M. G. Celli

**Affiliations:** 1 Consejo Nacional de Investigaciones Científicas y Técnicas (CONICET) 62873, Buenos Aires, Argentina; 2 Facultad de Ciencias Agrarias (FCA-UNNE), Corrientes, Argentina; 3 PHIM, Univ Montpellier, IRD, INRAE, CIRAD, Institut Agro, Montpellier, France; 4 Unidad de Fitopatología y Modelización Agrícola (UFyMA, INTA-CONICET), Cordoba, Argentina; 5 Instituto de Patología Vegetal (IPAVE-CIAP-INTA), Córdoba, Argentina; 6 Estación Experimental Agropecuaria Corrientes (EEA Corrientes-INTA), Corrientes, Argentina; 7 Agencia Extensión Rural San Javier (AER San Javier-INTA), Santa Fe, Argentina; Katholieke Universiteit Leuven, Leuven, Belgium

**Keywords:** plant viruses, metagenomics, molecular epidemiology, phylogenetic analysis

## Abstract

While rice necrosis mosaic virus (RNMV) has only been described in Asia, we identified this virus on a rice plant from Argentina using a viral metagenomic approach. We further confirmed this result by RT-PCR and small-RNA Illumina sequencing to obtain the near-complete genome and to confirm actual infection by RNMV.

## ANNOUNCEMENT

**Fig 1:**
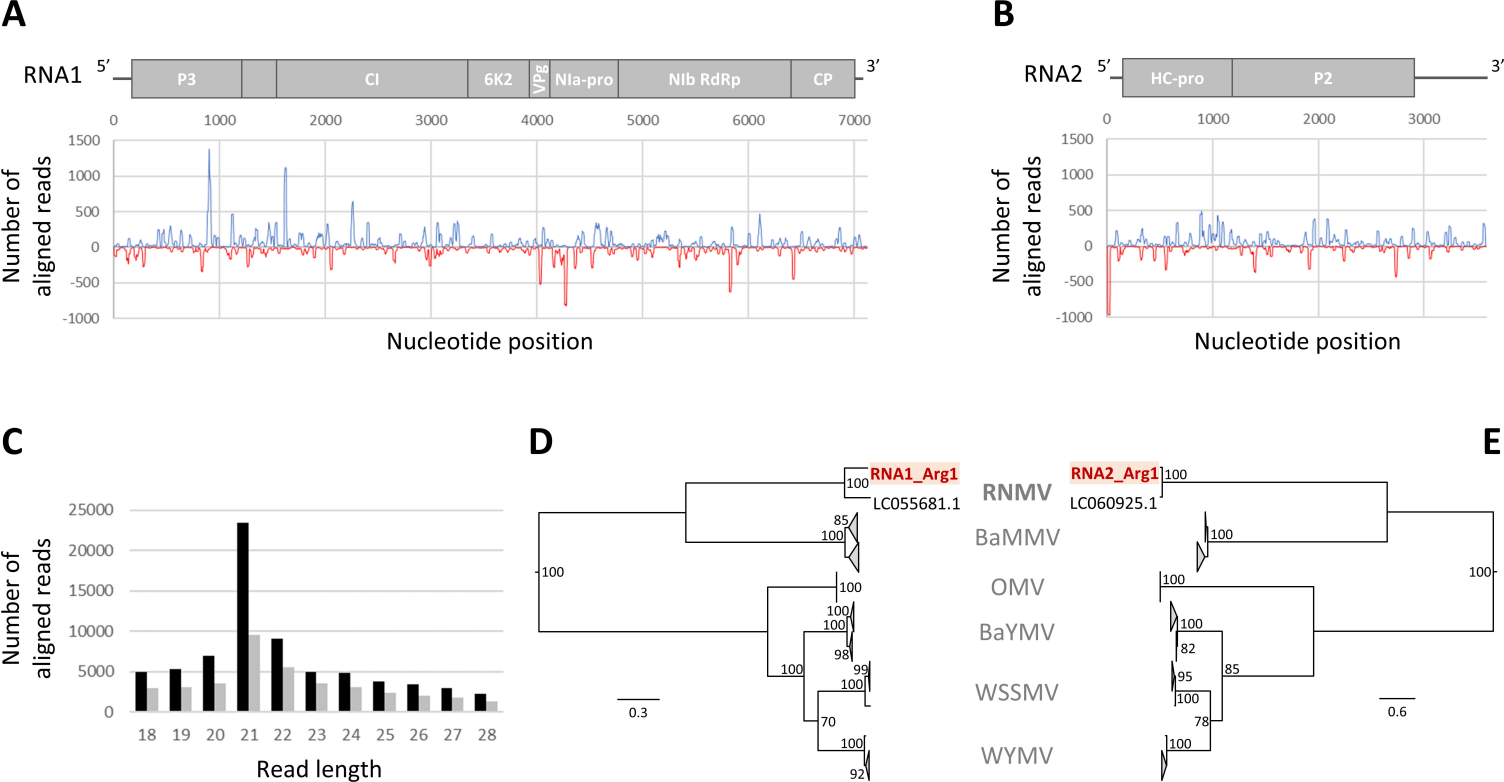
Genome organization of RNMV, main characteristics of viral small RNA (sRNA) isolated from the symptomatic rice plant sampled in Corrientes province (Argentina) and phylogenetic reconstructions. Number of small RNAs mapped to the RNMV reference genome distributed along RNA1 (**A**) and RNA2 (**B**), where the values above the axis represent sense (forward, in blue) reads starting at each position and those below represent antisense (reverse, in red) reads ending at the respective position. (**C**) Size distribution of RNA1 (black) and RNA2 (gray) RNMV genome mapping reads. Phylogenetic trees of RNA1 (**D**) and RNA2 (**E**) reconstructed with MEGA12 (10) by the maximum likelihood method with the best model (GTR + G) from the genomic sequences of 74 bymovirus isolates representing the genetic diversity of RNMV (species *Bymovirus oryzae*), barley mild mosaic virus (species *Bymovirus hordei*), oat mosaic virus (species *Bymovirus avenae*), barley yellow mosaic virus (species *Bymovirus hordeiluteum*), wheat spindle streak mosaic virus (species *Bymovirus tritici*), and wheat yellow mosaic virus (species *Bymovirus triticitessellati*).

Among the viruses reported to infect rice worldwide, only rice hoja blanca virus (species *Tenuivirus oryzalbae*, family *Phenuiviridae*), rice stripe necrosis virus (RSNV; *Benyvirus oryzae*, *Benyviridae*), and Mal de Rio Cuarto virus (*Fijivirus cuartoense*, *Spinareoviridae*) have been reported in South America (1, 2). In this study, we used a virion-associated nucleic acids, Illumina HiSeq (2×150-nucleotide paired-end reads) approach (3–5) on twelve symptomatic rice (*Oryza sativa*) plants collected in 2018 in the provinces of Corrientes and Santa Fe. A total of 3,780,924 trimmed reads generated by Illumina sequencing (from 34,418 to 580,084 reads per sample) were *de novo* assembled using CAP3 (6) and viral contigs were identified by BLASTn and BLASTx. We detected RSNV in two samples from plants with necrosis, wrinkled leaves, and panicle deformation. From one plant sample (Arg1) showing necrotic leaves and collected at Berón de Astrada (Corrientes), we obtained 87 contigs up to 1,946 nucleotides in size and sharing a mean of 93.4% nucleotide identity with the rice necrosis mosaic virus (RNMV; *Bymovirus oryzae*, *Potyviridae*) reference genome (LC055681.1 and LC060925.1 for RNA1 and RNA2, respectively) characterized in Japan (7). RNMV infection was confirmed after total RNA extraction with TRIzol Reagent (Thermo Fisher Scientific) according to the manufacturer’s protocol, RT-PCR using specific primers (RNMV-R1-F-5′ and RNMV-R1-R-3′ (8) and Sanger sequencing of the 613 bp amplicon. Small RNA (sRNA) library preparation (NEB Next Multiplex Small-RNA Library Prep Set) and Illumina sequencing (NovaSeq 6000 SP Reagent Kit, SE50) was performed by Novogene on the same RNA extraction. In the 18nt–28 nt size fraction, we obtained 25,326,985 trimmed reads, of which 110,304 reads (i.e. 0.44%) mapped to the RNMV reference genome with Geneious v.9.1 (Biomatters). *De novo* assembly was performed using Bowtie1.3.1 and Samtools1.18 to obtain the near-complete genome of the RNMV isolate Arg1 (RNA1: 7126 nucleotides, mean coverage of 285.9, 202.5 sense, and 83.5 antisense; RNA2: 3587nt, mean coverage of 364.7, 232.3 sense, and 132.4 antisense; Fig. 1A and B) characterized by a G + C content of 44.2% and 41.7% for RNA1 and RNA2. Among the 18–28nt sRNAs mapped to RNMV, a predominance of 21nt and 22nt sRNAs was observed (Fig. 1C), which is consistent with the production of small interfering (si)RNAs resulting from the gene silencing defense mechanism set up by plant cells in response to RNA virus infection (9).

The Arg1 genome showed high nucleotide (nt) and amino acid (aa) identities with RNMV (nt: 79.5% and 95.1%; aa: 93.7%, and 96.9%; for RNA1 and RNA2, respectively) and much lower with other species of the genus *Bymovirus* (nt: >61.4% and >65.4%; aa: >55.0% and >37.4%; for RNA1 and RNA2, respectively). These results were supported by phylogenetic reconstructions (Fig. 1D and E). Specifically, the coat protein of the Arg1 isolate shared respectively 81.4% and 94.7% nt identity with LC055681.1 and U95205, the only two RNMV sequences deposited in the GenBank database (7, 11), 91.3% and 99.0% at the aa level. These values exceed the species demarcation threshold established for the family *Potyviridae* (12). Thus, while RNMV has only been reported in Asia (1, 7, 11, 13), our results demonstrate that this virus is also present in rice fields in Argentina.

## Data Availability

The RNA1 and RNA2 complete sequences of the RNMV isolate Arg1 are deposited in GenBank under the accession no. PV583919 and PV583920, respectively (SRA accession: SRR34000318; Biosample accession: SAMN49111485).
